# The Late Bronze Age settlement site of Březnice: Magnetometer survey data

**DOI:** 10.1016/j.dib.2021.106793

**Published:** 2021-01-27

**Authors:** Martin Kuna, Roman Křivánek, Ondřej Chvojka, Tereza Šálková

**Affiliations:** a Institute of Archaeology of the Czech Academy of Sciences, Prague, Letenská 4, 118 01 Praha 1, Czech Republic; b Institute of Archaeology, University of South Bohemia in České Budějovice, Faculty of Arts, Branišovská 31a, 370 05 České Budějovice, Czech Republic

**Keywords:** Magnetometry, Source data, Bronze Age settlement site, Intra-site patterning, House burning, Prehistoric homestead clusters

## Abstract

The archaeological site of Březnice (Czechia) represents one of the large settlements of the Late Bronze Age (Ha A2/B1, 14C: 1124–976 BC) in Bohemia. The site became known mainly for a high number of so-called ‘trenches’, oblong pit features (breadth around 1 m, length 4–7 m), remarkable not only for their specific shape but also for their contents (unusual amounts of pottery, daub, loom weights and other finds, often with traces of a strong fire).

In 2018–20, a research project focusing on the study of the site was realized. Magnetometer survey became an integral part of the project since it represented a way to obtain an overall image of the site. A 5-channel fluxgate gradiometer from Sensys (Germany) was used; the vertical gradient of the Z component of the Earth magnetic field was measured. In total, the survey covered an area of over 17 hectares and included over 1.8 million measurements. Profiles were orientated from east to west and data taken bidirectionally (alternate lines in opposite directions), in a 0.5 × 0.2 m grid.

The site is extraordinary due to the fact that all archaeological features discovered so far belong to a single archaeological period (Late Bronze Age). This makes the acquired data set exceptional. It can be further used by archaeologists and geophysicists, both to create alternative models of the dynamics of prehistoric settlements and to better understand the nature and interpretive possibilities of the magnetometer data in archaeology as such.

## Specifications Table

**Table:** 

Subject	Social Sciences – Archaeology
Specific subject area	magnetometer survey data from the prehistoric site of Březnice (Czechia)
Type of data	SHP (GIS) feature files with attribute tables, XLXS file with secondary data and images/maps as illustrations
How data were acquired	on-site measurement with a fluxgate gradiometer followed by a GIS-based data analysis (cf. section Experimental Design, Materials and Methods)
Data format	raw (primary) magnetometer data after basic control and processing; list of polygons of magnetic anomalies (secondary data)
Parameters for data collection	research project on the settlement site of Březnice in 2018–20
Description of data collection	fieldwork of one of the authors (R. Křivánek) during several field campaigns (2018–20); subsequently, GIS analysis was performed by M. Kuna, classification criteria were discussed in the author team
Data source location	Institute of Archaeology CAS, Prague, v. v. i.; Prague, Czech Republic. The site is located in South Bohemia, Czechia, dist. Tábor; WGS84: N 49.2462, E 14.4938 (geographical coordinates in the S-JTSK system are part of the SHP files).
Data accessibility	https://doi.org/10.5281/zenodo.4453184
Related research article	Kuna, M. – Křivánek, R. – Chvojka, O. – Šálková, T., A quantitative approach to magnetometer survey data: the case of the Late Bronze Age site of Březnice, Journal of Archaeological Science 126 (2021) 105298

## Value of the Data

• An extremely large and coherent set of spatial data is provided, which may be used for further analysis of the prehistoric settlement structure as well as for the development of methods of classification and interpretation of magnetometer data.
• The data may be beneficial for geophysicists, archaeologists and students of both disciplines (archaeology and applied geophysics).
• The data can be subjected to different types of alternative evaluations to confirm or modify the results obtained so far. New ways of data analysis and interpretation can be tested.
• The data can also be used for training students.

## 1 Data Description

The data presented in this article are made up of two basic files (***01, 02***). Both files are in the SHP format, they were created in ArcGIS software.

The ***01_Breznice_data.SHP*** file contains raw data as measured in the field (followed by editing in the Magneto software, Sensys, and cleaning in the Surfer software). In this form and to this extent, the data (total of 1806,390 points) became the basis for further processing and analysis, described in the co-submitted research paper [1]. Relevant columns of the attribute table are:

• X, Y: coordinates in the S-JTSK system (https://epsg.io/5514–1623)
• Elevation: vertical gradient of Z component of the Earth magnetic field

The ***02_Breznice_anomalies.SHP*** file represents the set of polygons (magnetic anomalies) created by (i) the interpolation of the raw data into a GIS raster layer (grid 0.2 × 0.2 m) and (ii) cutting out of the surfaces with a value greater than +3 nT. These polygons were understood as magnetic anomalies relevant to archaeological analysis. Relevant columns of the attribute table are:

• Id: anomaly ID number, linking the polygon with the properties described in the ***03*** file
• plocha: area (in square meters)
• obvod: perimeter (meters)
• easting, northing: centroid coordinates (S-JTSK system)

The properties of individual magnetic anomalies were obtained in GIS by zonal analysis using the set of polygons in the ***02*** file and the interpolated layer of the raw magnetometer data. The values were saved in the file ***03_Breznice_anom_properties.XLSX*** file and used for statistical processing. Relevant columns of the table are:

• ID: link to ***02_Breznice_anomalies.SHP*** (anomaly ID number)
• COUNT_CELL: number of raster cells in the polygon (anomaly)
• MIN, MAX, RANGE, MEAN, STD, SUM: values (nT) calculated from the interpolated magnetometer data. These values were relevant for the classification of anomaly categories (individual measurements can be obtained from the 01 and 02 SHP files).

Files ***04*** and ***05*** contain additional information for the display and analysis of data in a GIS environment. The ***04_Breznice_masque.SHP*** file displays the total extent of the magnetometer survey in 2018–20; the survey was conducted in two large polygons (agricultural fields) separated by a road.

The ***05_Breznice_excavations.SHP*** file shows the position of the excavation trenches from 2005–9 and test trenches from 2019. This information may be important to determine the areas where magnetometer values are either not relevant (disturbed by the preceding excavation) or, on the contrary, usable for the comparison of the magnetometer data with the results of archaeological excavation (test trenches from 2019). We do not publish, however, the location of individual archaeological features discovered by the excavation here, because the data is still being processed and prepared for publication; moreover, it is not any important issue of our research paper. In case of any research interest, this data is available on request. Relevant columns of the attribute table:

• sonda: name/number of the excavation trench
• EXC_GF: two attribute states – ‘after GF’ (excavation followed the magnetometer survey), empty field (excavation preceded magnetometer survey; the magnetometer data are distorted)

The following files are illustrations documenting the site and the results of the magnetometer survey. These files do not require any further comment or explanation.

*06_Breznice_orthophoto.JPG*: location of the site (polygon of magnetometer survey) on an orthophoto map. X, Y coordinates are given in the S-JTSK system (https://epsg.io/5514–1623).

*07_Breznice_colour.JPG*: interpolated magnetogram in a colour scale. Min: −6 nT, max: +6 nT.

*08_Breznice_greyscale_1_6.JPG*: interpolated magnetogram in a grey scale. Min: −1 nT, max: +6 nT.

*09_Breznice_greyscale_4_4.JPG*: interpolated magnetogram in a grey scale; min. −4 nT, max. +4 nT.

## 2 Experimental Design, Materials and Methods

The magnetometer survey in Březnice covered the area of 17.3 ha (1806.390 points). A 5-channel fluxgate gradiometer mounted on a two-wheeled pushcart from Sensys (Germany) was used. The vertical gradient of the Z component of the Earth magnetic field was measured, profiles were orientated from east to west and data taken bidirectionally (alternate lines in opposite directions), in a 0.5 × 0.2 m grid.

Raw magnetometer data (***01***) was converted into GIS and interpolated into a raster image with a 0.2 × 0.2 m grid (using the Natural Neighbour method, ArcGIS). Surfaces exceeding +3 nT were cut out and included into the file ***02***. The main statistical variables for individual polygons were calculated in GIS (Zonal Statistics as Table procedure) and stored in the ***03*** file.

Three files presented here (***01, 02, 03***) contain all the data that was used for our analysis of the Late Bronze Age settlement site in Březnice. The analysis itself and its results are described in the co-submitted research paper [1]. Complete magnetometer data from our survey are made accessible to other research projects that could confirm or modify our conclusions.

## Ethics Statement

The authors are not aware of any controversial issues regarding publication ethics.

## CRediT Author Statement

**Martin Kuna:** Conceptualization, Methodology, Formal analysis, Visualization and Writing; **Roman Křivánek:** Resources and Methodology; **Ondřej Chvojka:** Resources and Project administration; **Tereza Šálková:** Resources.

## Declaration of Competing Interest

The authors declare that they are not aware of competing financial interests or personal relationships, which have or could be perceived as having influenced the work reported in this article.
